# Pre-Pandemic Predictivity of Anxious-Depressive Symptoms in Post-Surgical Traumatic Distress in Hysterectomy for Benign Disease and COVID-19 Outbreak: A Case-Control Study

**DOI:** 10.3390/jcm13113148

**Published:** 2024-05-27

**Authors:** Marta Ielmini, Jvan Casarin, Camilla Callegari, Alessandro Bellini, Manuela Giada Favata, Anna Giudici, Fabio Ghezzi, Antonella Cromi, Ivano Caselli

**Affiliations:** 1 Department of Medicine and Surgery, Division of Psychiatry, University of Insubria, 21100 Varese, Italy; marta.ielmini@uninsubria.it (M.I.); camilla.callegari@uninsubria.it (C.C.); mgfavata@studenti.uninsubria.it (M.G.F.); 2 Obstetrics and Gynecology Department, University of Insubria, 21100 Varese, Italy; jvan.casarin@uninsubria.it (J.C.); agiudici6@studenti.uninsubria.it (A.G.); fabio.ghezzi@uninsubria.it (F.G.); antonella.cromi@uninsubria.it (A.C.); 3 Department of Applied and Psychobehavioral Sciences, Division of Psychiatry, University of Pavia, 27100 Pavia, Italy; a.bellini2@uninsubria.it

**Keywords:** post-traumatic stress disorder, post-surgical stress, anxiety, depression, hysterectomy, COVID-19

## Abstract

**Background:** The severe acute respiratory syndrome coronavirus (SARS-CoV-2) pandemic led to several needed containment measures that conditioned the onset of depressive, anxiety, and post-traumatic stress symptoms in the population. These symptoms, especially if not diagnosed and treated, can also occur in patients undergoing medical care or surgery, with a high impact on people’s lives and causing low adherence to treatment. The study evaluates whether the spread of the coronavirus disease 2019 (COVID-19) worsened the onset of post-surgical distress and symptoms of anxiety and depression in a population undergoing hysterectomy for benign disease during the pandemic era, comparing it with a population with the same characteristics but recruited before COVID-19. **Methods:** The sample was evaluated before surgery (T1), post-operatively (T2), and 3 months after surgery (T3) through a sociodemographic questionnaire and through the HADS (Hospital Anxiety and Depression Scale) to evaluate anxious-depressive symptoms and the PCL-5 (Post-traumatic Stress Disorder Checklist for DSM-5) to assess the onset of post-surgical distress. **Results:** Patients treated after the COVID-19 pandemic showed a higher depressive symptoms rate compared with those treated before (*p*-value = 0.02); conversely, pre-COVID-19 patients were more prone to develop post-traumatic stress disorder (PTSD) (*p*-value = 0.04). A significant association between the occurrence of PTSD and anxiety-depressive symptoms registered at T2 (*p*-value = 0.007) and T3 (*p*-value < 0.0001) emerged. In the end, the COVID-19 pandemic has exerted a detrimental influence on the mental well-being of the patients under investigation, with a notable exacerbation of their mood disturbances. **Conclusions:** The findings advocate for the implementation of psychometric and psychodiagnostic assessments to promptly detect high-risk scenarios that could lead to PTSD, compromising treatment compliance and exacerbating the overall outcome, resulting in substantial direct and indirect burdens.

## 1. Introduction

In the last decades, the interest in the psychophysical integrity of surgical patients and in the understanding of post-operative distress in the context of healthcare events has increased [1], and growing interest has been directed to specific clusters of symptoms, usually defined as post-traumatic stress disorder (PTSD), but determined by medical or surgical procedures. The incidence of this disturbance ranges from 12% to 25% among patients who have undergone various medical or surgical treatments [2]. This was corroborated by a previous study involving patients undergoing surgery for benign gynecological conditions, showing that 16.4% of patients experienced PTSD at the three-month follow-up, with no correlations with sociodemographic or gynecologic characteristics. Furthermore, a significant association between depression (HADS > 8) and PTSD symptoms during the post-surgical period (*p*= 0.002) and at a three-month follow-up (*p* < 0.001) was shown [3], which is in line with the current literature. This form of post-surgical PTSD differs from the conventional PTSD as defined by DSM-5 (Diagnostical and Statistical Manual of Mental Disorder, Fifth Edition), primarily due to differences in the source of threat, with external factors versus somatic origins, as well as distinctions in the cognitive aspects of threat related to the past, present, or future. Furthermore, there are variations in the avoidance behaviors displayed and the nature and consequences of hyperarousal, which are typically linked to post-surgical pain-induced PTSD. However, both disorders share a key similarity: the fear of death [3,4]. This specific disorder appears to be associated with unfavorable outcomes, manifested through poor adherence to therapies and follow-up appointments, as well as an elevated risk of complications. Moreover, the literature describes frequent associations between post-surgical PTSD and depressive and anxious symptoms. As reported above, a previous work evaluated the incidence of post-surgical PTSD after hysterectomy due to benign gynecological disease since hysterectomy represents the most common gynecological procedure in developed countries [2]. Unaddressed and untreated manifestations of anxiety and depression after hysterectomy for benign conditions can exert detrimental effects on morbidity management, adherence to treatment protocols, disease recovery, and overall quality of life [5]. On the other hand, the treatment of PTSD can be really tough due to the lack of effective pharmacotherapy [6], which is different from psychopharmacotherapy for other psychiatric disorders [7]. Since 2020, the severe acute respiratory syndrome coronavirus (SARS-CoV-2) pandemic has had a detrimental impact on people’s mental health, affecting not only infected individuals but also healthy people and patients hospitalized for other reasons. This can be attributed to factors such as being separated from loved ones, loss of personal freedoms, uncertainty surrounding one’s health, and potentially experiencing boredom [8]. These circumstances have significantly influenced and exacerbated the psychological well-being of the general population. The emergence of the COVID-19 pandemic has created an environment where many factors of poor mental health have been exacerbated. Notably, in the literature, the most commonly described psychological conditions during this period among the general population include negative emotions, such as sadness and loneliness, anxiety, depressive symptoms, post-traumatic stress symptoms [9], irritability, panic attacks, phobic symptoms, insomnia, fits of anger, and emotional exhaustion [8]. In many cases, patients who had already contracted the SARS-CoV-2 infection also experienced an intense fear of reinfection [10] with consequent avoidance of places of treatment and social situations. Referring to infected people, Deng and colleagues highlighted that 45% of COVID-19 patients experienced depression, 47% of patients experienced anxiety, and 34% of patients experienced sleep disturbances [8]. As described in the literature, the pandemic led to a 27.6% increase in cases of major depressive disorders and a 25.6% increase in cases of anxiety disorders globally [9].

Another consequence related to the fear of contagion was the healthcare avoidance behavior with the worsening of pre-existing diseases or delayed diagnosis of unknown conditions. In this panorama, this study focused on the role of the pandemic context in exacerbating symptoms such as post-surgical PTSD, anxiety, and depression in a specific population of patients hysterectomized for benign pathology. A strong point of the study was the possibility of comparing two homogeneous samples in terms of clinical and sociodemographic characteristics that were subjected to the same surgical procedure but different in terms of the recruitment period, i.e., pre and post COVID.

Assuming that SARS-CoV-2 has worsened the mental well-being of the general population, the study focuses on hospitalized patients. Specifically, the present study aimed to compare data of patients who underwent hysterectomy for benign gynecological disease in a pre-COVID-19 period (January–May 2019) and data collected during the COVID-19 pandemic (April–September 2021). Specifically, the two groups of patients were compared using psychometric instruments in terms of the following:

- Post-surgical traumatic distress in the early post-surgical period (T2) and three months later;
- The onset or worsening of anxiety and depressive symptoms in the post-surgical period (T2) and three months later (T3).

## 2. Materials and Methods

### 2.1. Study Design

**Figure 1:**
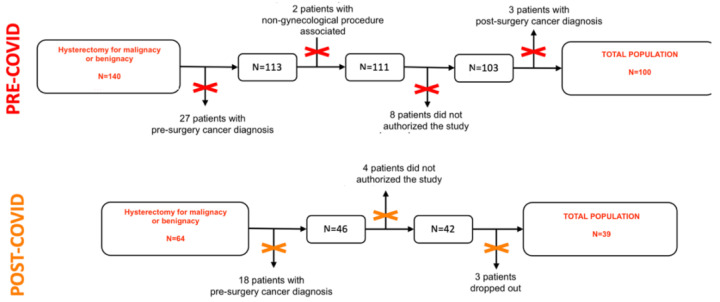
Populations flowchart.

This is a prospective observational cohort study that involves a comparison of two distinct populations. The sample size was estimated based on previous studies on PTSD following non-gynecologic surgery [10]. The first group consisted of 100 subjects who were recruited consecutively before the onset of the COVID-19 pandemic, from April 2021 to September 2021, while the second group included 39 subjects who were recruited after the pandemic began, from January to May 2019 (Figure 1). All participants in both groups had a medical indication for hysterectomy due to benign disease and were referred to the Obstetrics and Gynecologic Department of the Women’s and Children’s Hospital of Varese (Filippo Del Ponte Hospital, Varese, Italy), where the surgery was performed. Prior to the participation, all individuals were duly informed about the study’s objectives and procedures and provided written consent for the use and analysis of the data.

### 2.2. Inclusion and Exclusion Criteria

To be recruited, patients had to fulfill the following inclusion criteria:

- Female sex;
- Age ≥ 18 years;
- To be a patient of the Gynecologic Department of Women’s and Children’s Hospital of Varese (Filippo Del Ponte Hospital, Varese, Italy);
- To sign a written informed consent for the use of anonymous data for scientific scope;
- To undergo total hysterectomy for the following benign disease: fibromatosis; abnormal uterus bleeding; endometriosis/adenomyosis; pelvic/abdominal pain; genital prolapse; and uterus (endometrial hyperplasia) and cervical intraepithelial cervical cancer (precancerous lesions).

Patients were excluded from the study based on the following exclusion criteria:

- No Italian language understanding;
- Association of hysterectomy with other non-gynecological interventions;
- Gynecological malignancy or secondary metastasis;
- To be affected by neuropathic/chronic pain;
- Taking anti-inflammatory drugs;
- Enlistment in other studies with pharmacological intake;
- To have a current diagnosis of a major psychiatric disorder.

Patients with psychiatric disorders and subjects unable to understand the Italian language were excluded to avoid confounding variables. The table below summarizes the inclusion/exclusion criteria (Table 1).

Patients were evaluated at three different times through a social-demographic questionnaire (age, marital status, employment, instruction, pharmacological therapy, and parity) and through the administration of the psychometric instruments, as shown in Figure 1.

For the psychopathological evaluation, two validated questionnaires were administered to patients: the Hospital Anxiety and Depression Scale (HADS) and the Post-traumatic Stress Disorder Checklist for DSM-5 (PCL-5).

The HADS is a widely used screening instrument with seven anxiety and seven depression items. The scores of the anxiety and depression subscales range from 0 to 21. A total score of <8 was used to establish the absence of symptoms for both anxiety and depression [11].

The PCL-5 scale was used to assess PTSD symptoms. This 20-item questionnaire was updated in 2013 and is the most used questionnaire to evaluate PTSD symptoms following surgical procedures. It allows to calculate a total symptom severity score (obtained by summing the scores for each of the twenty items) ranging from 0 to 80.

The final score was calculated by adding up the scores of each cluster (a provisory diagnosis of PTSD can be diagnosed with a minimum score of one item in clusters B and C and two items in clusters D and E) [12]. The PLC-5 has not been translated into Italian; thus, it was administered in English. In instances where the participant faced difficulty in comprehension, the scale was presented through an interview conducted by a specialized operator. Both questionnaires are provided as Supplementary Materials.

### 2.3. Research Timeline

The questionnaires were administered according to the following timeline, as shown in Figure 1:

- T1: pre-surgery consulting by a gynecologist from one month to one week before the interventions. Here, patients were asked to join the study. Those who accepted had to fill out a social-demographic questionnaire and the HADS scale;
- T2: first post-surgery day. Patients had to fill out the HADS and PCL-5 scales;
- T3: three months after surgery. Each patient was visited at the outpatient service or was called to administer the HADS and PCL-5 scales.

Patients were evaluated at an outpatient clinic, and the assessment was performed by clinicians and residents in T1 and in the T3 follow-up period. T2 evaluations were performed by clinicians and residents during the hospitalization. At the T3 evaluation, if it was impossible to visit patients in person, they were contacted by telephone.

In both populations, the following data were also collected: the primary indication of hysterectomy (symptomatic uterine fibromatosis, abnormal uterus bleeding, endometriosis/adenomyosis, pelvic/abdominal pain, genital prolapse, and precancerous lesions), surgical data (surgical approach, removal of the ovaries), pathology report (Division of Pathologic Anatomy and Histology of Circolo and Macchi Foundation Hospital of Varese, Varese, Italy), and post-operative complications (including ER accesses).

### 2.4. Statistical Analysis

Data were analyzed using the software SPSS Inc. (released software 2015, IBM SPSS statistics for Mac, Armonk, NY, USA) [13]. Descriptive analyses were reported as absolute numbers and percentages (%). D’Agostino and Pearson test was used to evaluate the normal distribution of data. The unpaired *t*-test and the Fisher exact test were used to assess differences between groups at baseline for continuous and dichotomous variables. Significance for all tests was set at *p*≤ 0.05, two-tailed.

The study was conducted by taking into account regulatory requirements and legal requirements (DL n.211, 24 June 2003, and DM 17 December 2004) and in accordance with the ethical principles of the Declaration of Helsinki.

## 3. Results

### 3.1. Sociodemographic Characteristics of the Samples

The flowchart of the study population is reported in Figure 1. Overall, the study focused on 139 patients enrolled in the investigation: 100 (71.9%) in the pre-COVID-19 cohort and 39 (28.1%) in the post-COVID-19 cohort. Baseline sociodemographic features of both groups are shown in Table 2.

The two groups did not differ in sociodemographic and clinical characteristics at the baseline. The median age at the time of surgery was 51 years (SD 7.2) for the pre-COVID group and 47 years (SD 6.5) in the post-COVID group, without a significant difference between the groups. In both populations, the patients demonstrated a good level of education, with 50% and 51% of the samples having finished high school in the pre- and post-COVID groups, respectively. Furthermore, the majority of individuals in both samples were employed (73% and 74%, respectively, in the pre-COVID-19 and post-COVID-19 groups), the majority of patients were married (64% and 72%, respectively, in the pre-COVID and post-COVID groups), most patients had at least one child, lived with family, and the majority of patients in both groups did not undergo psychopharmacotherapy (80% and 8%, respectively, in the pre-COVID and post-COVID groups). Surgical-related outcomes of the two groups are reported in Table 3.

No substantial disparities were observed in terms of surgical approach; laparoscopy emerged as the predominant surgical method in both groups, with rates of 90% and 95% in the pre-COVID and post-COVID groups, respectively. Similarly, there were no discernible distinctions in the indications for surgery, with uterine fibroids constituting the most prevalent indication in both cohorts. Furthermore, the majority of treated patients did not undergo oophorectomy beyond hysterectomy. Notably, a majority of the patients did not encounter post-operative complications related to surgery, with rates of 90% and 82% in the pre-COVID and post-COVID groups, respectively.

### 3.2. Psychopathological Features of the Populations

Depressive and anxiety trend evaluations at T1, T2, and T3 are shown in Table 4. Differences in the psychopathological evaluation were highlighted at T1, T2, and T3.

Regarding the anxiety score, no statistically significant differences were found between the two groups at T1, T2, or T3.

In terms of depressive scores, a statistically significant difference in total scores between the two populations was observed at T1 (*p*-value < 0.001). Specifically, the post-COVID-19 population exhibited higher rates of mild and severe symptoms (*p*-value = 0.05) and general depressive symptoms (*p*-value = 0.02). A higher proportion of women displayed depressive symptoms in the post-COVID-19 sample compared with the pre-COVID-19 sample (*p*-value = 0.02). No statistically significant differences were found between the two groups at T2. However, a statistically significant difference (*p*-value = 0.01) in mood deflection at T3 favored the pre-COVID-19 population.

**Figure 2:**
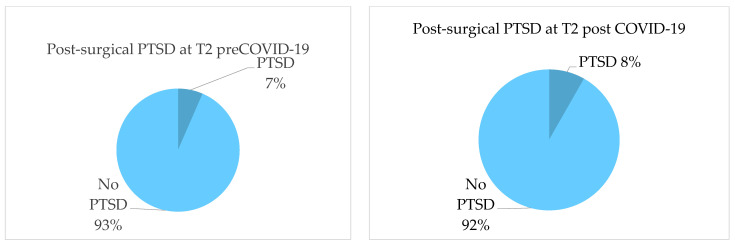
Distribution of post-surgical PTSD over time (T2).

**Figure 3:**
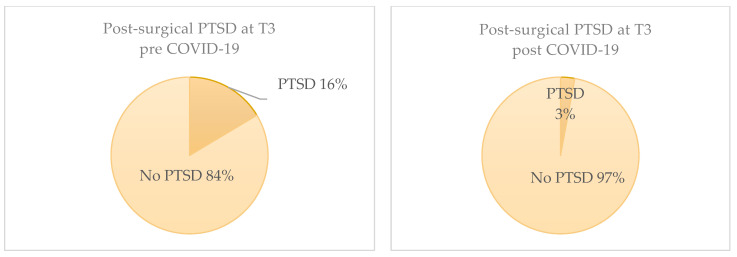
Distribution of post-surgical PTSD over time (T3).

Regarding PTSD symptoms, 7% in the pre-COVID-19 population and 8% in the post-COVID-19 population showed symptoms referable to PTSD with no statistically significant differences at T2 (*p*-value = 1) (Figure 2). At T3, PTSD symptoms were statistically significantly higher in the pre-COVID-19 population, with 14 patients (16.4%) vs. one patient (3%) (*p*-value =0.04) (Figure 3).

A total of 15 patients were diagnosed with PTSD (14 subjects in the pre-COVID-19 period and one woman in the post-COVID-19 period). The remaining 109 patients (73 pre-COVID-19 and 36 post-COVID-19) did not meet the criteria for a PTSD diagnosis.

The only significant factor among all the sociodemographic and surgical variables associated with PTSD was the recruitment period; specifically, we found a positive association between the pre-COVID-19 period and PTSD (*p*-value = 0.03). PTSD at T3 was identified in 16% of patients during the pre-COVID period (N = 14) compared with 3% in the post-COVID period.

## 4. Discussion

The paper focused on the evaluation of the impact of the pandemic era on the mental health of hospitalized patients, particularly investigating any influence of the pandemic context in the onset of post-surgical PTSD and anxiety and depressive symptoms in the post-operative period. The aim was reached through the comparison between a group composed of patients hysterectomized for benign pathology recruited before the spread of COVID-19 and a sample with the same characteristics recruited during the pandemic era. The aim was to detect any difference in terms of post-surgical distress and anxiety and depression symptoms by comparing two similar samples that are homogeneous in sociodemographic and clinical characteristics. This evaluation is part of a line of research aimed at understanding the global psychophysical well-being of patients undergoing gynecological surgery. As mentioned above, the COVID-19 pandemic had various consequences that involved all aspects of people’s daily lives, starting from social life up to the economic system. Its clinical implications concern not only short-term complications but also medium and long-term consequences, which are partially known. Psychological and psychiatric sequences can be observed in both psychiatric and non-psychiatric populations. In particular, the fear of contagion from SARS-COVID-19 can worsen an experience such as that experienced by patients who undergo oncological surgery, leading to the onset of post-traumatic stress symptoms or disorders [9]. The onset of this disorder can seriously affect adherence and prognosis. Depressive and anxious symptoms immediately follow the onset of PTSD, both in psychiatric and non-psychiatric patients [3,8], leading to lower adherence to treatment and avoidance of follow-up. The normal course of these symptoms can be further complicated by the fear of contagion and its consequences, which leads people to almost completely avoid places of treatment.

Our results were partially in agreement with the initial hypothesis. Post-surgical distress was prevalent in the pre-pandemic population, affecting 14.6% of the tested population, which is in line with the literature and with a lower percentage in the post-pandemic population.

The pandemic contest seems not to have worsened the onset of post-surgical PTSD, resulting in being more frequent in the pre-pandemic period. Otherwise, the COVID-19 population showed a higher level of depression than the pre-pandemic era population. The trend of depression showed a decrease during the study period in both populations, but the sample recruited during COVID-19 showed a higher level of depression, in line with the literature [14,15,16].

Otherwise, with regard to anxious symptomatology, no differences emerged in the two groups, which showed a decreasing trend from the pre-operative evaluation to the three-month follow-up evaluation. Also, in the literature, anxiety in the perioperative period tends to have a decreasing trend, with particularly high values in the immediate pre-operative period and with a decreasing trend in the post-operative period, especially when communication between patient and clinicians is clear and exhaustive and when there are no post-surgical complications [17].

A previous study performed by our research team concluded that hysterectomy for benign disease is associated with a non-negligible risk of post-surgical PTDS; the use of the HADS questionnaires might be useful to select patients who might benefit from dedicated psychological support [2]. In this work, a relevant correlation emerged between depressive symptoms and post-traumatic stress disorder. In fact, 40% of women with post-surgical depression developed PTSD in the follow-up, and 80% of patients with depressive symptoms in T3 had comorbid post-traumatic symptoms. For these reasons, we encourage the use of psychometric and psychodiagnostic scales to identify early these risks that could evolve into PTSD, compromising therapeutic adherence and worsening the outcome with consequent direct and indirect costs [18].

Depressive symptoms in the pre-COVID-19 population without basal depressive symptoms show a statistically significant progression. Mood deflection is the only variable predicting PTSD onset. A total of 40% of the patients with depressive symptoms developed PTSD at T2, while 30% of them developed it at T3. Three months away from surgery, the PTSD rate in the population was 3% in the post-COVID-19 population and 16% in the pre-COVID-19 population.

The association between PTSD and surgical characteristics showed no significant relevance. The only variable that was statistically significant was the period of recruitment. A total of 93% of patients in the pre-COVID-19 period developed PTSD three months after surgery.

This result can be explained by the fact that people in the post-pandemic period tended to relocate the subject of concern from the surgery to the pandemic situations and its consequences, experiencing the end of the hospitalization as the end of the extreme distance from family members and benefiting from the idea of being discharged without contracting the COVID-19 infection [19,20].

Moreover, the results showed that the COVID-19 pandemic negatively impacted the mental health of the evaluated patients, especially worsening their mood. This result is confirmed by other studies in the professional literature [21,22]. Post-surgery distress decreased during the pandemic era (*p*-value = 0.04), maybe due to the fear of getting COVID-19 in the hospital, which led to desert clinical check-ups [23].

As far as we know, no studies have investigated the correlation between anxious and depressive symptoms in post-surgical stress consequent to benign disease in different clinical settings and different populations. More studies are needed to explore this correlation and its clinical implications. This study highlights both the importance of early detect the onset of anxious and depressive symptoms and post-surgical PTSD after hysterectomy and the negative effects of the COVID-19 pandemic, indicating the potential need for support services for women [24].

Some limitations need to be addressed. First, the study includes a relatively small sample size, particularly in the post-COVID-19 group. A limitation due to the possible diversity of the two samples has to be considered. Secondly, we employed a self-administered psychometric scale that indicates the presence of symptoms but does not enable a diagnostic determination. Third, all the operations were performed via minimally invasive surgery, specifically laparoscopy, which did not allow for the evaluation of the impact of other surgical approaches on PTSD. The assessment of the level of education faced the same limitation, as all the patients reported having a good level of education. A further limitation of the study is that it is not possible to provide data on long-term outcomes. Based on the limits of the study, future perspectives research includes extending the study by comparing the onset of post-surgical PTSD and anxiety and depression between laparoscopic and laporotomic approaches. Another future goal is to compare patients undergoing hysterectomy for cancer with those for benign disease.

## 5. Conclusions

In conclusion, this study is part of a growing focus on the global well-being of surgical patients, allowing for a more regular post-operative course, reducing complications and increasing adherence rates to correct follow-up. Post-surgical distress could range on a spectrum from subsyndromal to syndromal post-traumatic stress disorder (PTSD), the object of our evaluation, causing relevant consequences to surgical patients. In a research line focusing on it, we have added a spotlight on the impact of the pandemic contest on the mental well-being of surgical patients.

Our results reveal how the pandemic era had a negative impact on the mental health of the patients who were assessed to suffer from depression, in line with current literature. On the other hand, post-surgical distress significantly decreased in the post-COVID-19 population. Our findings highlight the importance of early identification using psychometric and psychodiagnostic scales, as untreated cases may compromise therapeutic adherence and lead to adverse outcomes with associated direct and indirect costs.

The results of this investigation might contribute to a more comprehensive understanding of the observed correlations and facilitate the development of targeted interventions for at-risk individuals.

## Figures and Tables

**Table 1:** Inclusion and exclusion criteria.

Inclusion Criteria	Exclusion Criteria
Female sex	No Italian language understanding
Age ≥ 18 years old	Association with other non-gynecological interventions
Hospitalized in the Gynecologic Department in Filippo Del Ponte Hospital	Gynecological malignancy or secondary metastasis
Written informed consent signed	Neuropathic/chronic pain
Underwent a total hysterectomy for benign disease	Use of anti-inflammatory drugs
	Enlistment in other studies with pharmacological intake
	Have a current diagnosis of a major psychiatric disorder

**Table 2:** Sociodemographic variables in both populations.

Sociodemographic Variables	Pre-COVID-19 (N = 100)	Post-COVID-19 (N = 39)	*p*-Value
**Age**	51 (36–77)	47 (36–71)	0.10
<40	4 (4%)	4 (10%)	
40–50	45 (45%)	24 (62%)	
>50	51 (51%)	11 (28%)	
**Menopause**			0.44
Yes	27 (27%)	14 (36%)	
No	73 (73%)	25 (64%)	
**Education**			0.14
Primary	0 (0%)	2 (5%)	
Secondary	33 (33%)	10 (26%)	
High school	50 (50%)	20 (51%)	
Degree	17 (17%)	7 (18%)	
**Employment**			0.75
Employee	73 (73%)	29 (74%)	
Unemployed	16 (16%)	5 (13%)	
Housewife	9 (9%)	3 (8%)	
Retiree	2 (2%)	2 (5%)	
**Marital status**			0.72
Married	64 (64%)	28 (72%)	
Unmarried	22(22%)	7 (18%)	
Divorced	12 (12%)	4 (10%)	
Widowed	2 (2%)	0 (0%)	
**Parity**			0.11
0	29 (29%)	6 (16%)	
1	22 (22%)	13 (33%)	
>1	49 (49%)	20 (51%)	
**Living with**			0.59
Alone	15 (15%)	3 (8%)	
Family	77 (77%)	34 (87%)	
Parents	3 (3%)	1 (2.5%)	
Other	5 (5%)	1 (2.5%)	
**Pharmacological therapy**			0.46
No therapy	80 (80%)	34 (87%)	
Therapy	20 (20%)	5 (13%)	
Antidepressants	4 (4%)	0 (0%)	
Anxiolytics/hypnotic	14 (14%)	5 (13%)	
Combined	2 (2%)	0 (0%)	

**Table 3:** Surgical gynecological variables in both populations.

Surgery	Pre COVID-19 (N = 100)	Post COVID-19 (N = 39)	*p*-Value
**Surgical indication**			0.20
Fibromatosis	71 (71%)	28 (72%)	
Endometriosis	10 (10%)	5 (13%)	
Genital prolapse	14 (14%)	2 (5%)	
Abnormal bleeding uterus	3 (3%)	1 (2.5%)	
Endometrial hyperplasia	2 (2%)	1 (2.5%)	
Other	0 (0%)	2 (5%)	
**Surgical approach**			0.06
Laparoscopic	90 (90%)	37 (95%)	
Laparotomy	1 (1%)	2 (5%)	
Others	9 (9%)	0 (0%)	
**Oophorectomy**			0.21
Yes	25 (25%)	14 (36%)	
No	75 (75%)	25 (64%)	
**Post-surgery complications**			0.25
Yes	10 (10%)	7 (18%)	
No	90 (90%)	32 (82%)	

**Table 4:** Depressive and anxiety trend evaluations at T1, T2, and T3.

	Pre-COVID-19		Post-COVID-19	
	Median (Range)	*p*-Value	Median (Range)	*p*-Value
**HADS Anxiety Score**		0.01		0.02
T_1_	7 (0–21)		6 (0–16)	
T_2_	6 (0–18)		5 (0–18)	
T_3_	4 (0–20)		5 (0–12)	
**HADS Depression Score**		0.20		0.66
T_1_	3 (0–14)		5 (0–16)	
T_2_	3 (0–15)		4 (0–16)	
T_3_	2 (0–17)		4 (0–14)	
**HADS Depression Score**		<0.001		0.56
**Negative T_1_ cut-off**				
T_1_	3 (0–7)		4 (0–8)	
T_2_	3 (0–12)		3 (0–9)	
T_3_	4 (0–15)		3 (0–8)	
**HADS Depression Score**		0.10		0.02
**Positive T_1_ cut-off**				
T_1_	13 (8–21)		10 (8–12)	
T_2_	11 (5–18)		7 (2–16)	
T_3_	7 (0–20)		7 (0–14)	

HADS: Hospital Anxiety and Depression Scale.

## Data Availability

https://www.preprints.org/manuscript/202404.0274/v1.

## Supplementary Materials

The following supporting information can be downloaded at: https://www.mdpi.com/article/10.3390/jcm13113148/s1, Hospital Anxiety & Depression Scale (HADS) and PTSD Checklist for DSM-5 (PCL-5) [12].
